# The Role of Mitophagy in Regulating Cell Death

**DOI:** 10.1155/2021/6617256

**Published:** 2021-05-18

**Authors:** Sunao Li, Jiaxin Zhang, Chao Liu, Qianliang Wang, Jun Yan, Li Hui, Qiufang Jia, Haiyan Shan, Luyang Tao, Mingyang Zhang

**Affiliations:** ^1^Department of Forensic Sciences, School of Basic Medicine and Biological Sciences, Affilated Guangji Hospital, Soochow University, Suzhou, China; ^2^Department of Orthopedics, The Second Affiliated Hospital of Soochow University, Suzhou, China; ^3^Department of Obstetrics and Gynecology, The Affiliated Suzhou Hospital of Nanjing Medical University, Suzhou, China

## Abstract

Mitochondria are multifaceted organelles that serve to power critical cellular functions, including act as power generators of the cell, buffer cytosolic calcium overload, production of reactive oxygen species, and modulating cell survival. The structure and the cellular location of mitochondria are critical for their function and depend on highly regulated activities such as mitochondrial quality control (MQC) mechanisms. The MQC is regulated by several sets of processes: mitochondrial biogenesis, mitochondrial fusion and fission, mitophagy, and other mitochondrial proteostasis mechanisms such as mitochondrial unfolded protein response (mtUPR) or mitochondrial-derived vesicles (MDVs). These processes are important for the maintenance of mitochondrial homeostasis, and alterations in the mitochondrial function and signaling are known to contribute to the dysregulation of cell death pathways. Recent studies have uncovered regulatory mechanisms that control the activity of the key components for mitophagy. In this review, we discuss how mitophagy is controlled and how mitophagy impinges on health and disease through regulating cell death.

## 1. Introduction

**Figure 1:**
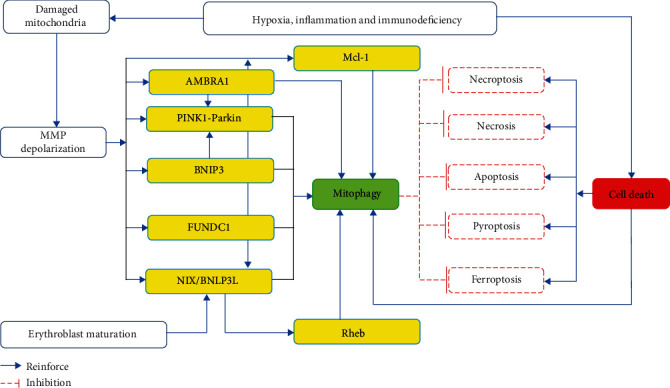
Mechanism of mitophagy in different physiological and pathological contexts and mitophagy regulating cell death pathway. Mitophagy plays an important role in maintaining mitochondria homeostasis and various aspects of cellular function. Under physiological conditions, NIX-mediated mitophagy is required for mitochondrial removal during erythroblast differentiation. When in response to mitochondrial stressors, such as membrane depolarization, mitochondrial complex dysfunction, mutagenic stress, and proteotoxicity, distinct mitophagy pathways cooperate to regulate mitochondrial homeostasis. Nix can interplay with Rheb, an important protein for mitophagy, to initiate mitophagy. Nix also functions downstream of Parkin as a substrate. In addition, BNIP3 is able to inhibit PINK1 proteolytic degradation to facilitate Parkin mitochondrial recruitment and mitophagy. Upon mitochondrial depolarization, Mcl-1, as a mitophagy receptor, underwent rapid PINK1/Parkin-dependent polyubiquitination and degradation. AMBRA1-mediated mitophagy is also dependent on Parkin. These findings suggest that these pathways cooperate with each other to ensure efficient mitophagy. With overwhelming mitochondrial damage, the cell death pathway (necrosis, apoptosis, pyroptosis, ferroptosis and necroptosis) becomes dominant, and mitophagy could prevent the accumulation of dysfunctional mitochondria by regulating the cell death pathway.

Mitochondria, which are present in all the eukaryotic cells, are important organelles that can be seen under an optical microscope upon appropriately staining. Around 1890, German biologist Richard Altmann first discovered small rod-shaped and granular structures in animal cells. Benda named these structures as mitochondria in 1898, and within the next 100 years, researchers gradually discovered that mitochondria as unique organelles play an important role in producing the bulk of adenosine triphosphate (ATP) by the oxidative phosphorylation process recognized as the powerhouses of the cell [1, 2]. Besides, mitochondria are involved in the synthesis and production of other substances such as fatty acid, amino acid, heme, and iron-sulfur cluster. Mitochondria are also a signaling hub for both innate immunity and cell death [3]. In view of the importance of mitochondria in cells, any factor that induces mitochondrial damage can also cause damage to cells or the human body to a certain extent. Due to the fact that the mtDNA structure does not contain histone packaging and its replication follows asymmetric division, it has a high mutation rate, and mtDNA damage is the dominant source of mutation [4]. Moreover, too many or ectopic free radicals that are generated during the energy generation process can cause different degrees of damage to mitochondria. Widespread damage to mitochondria causes cells to die because they can no longer produce enough energy. For these reasons, mitochondria have evolved a variety of mitochondrial quality control mechanisms, which are constantly involved in biogenesis, mitochondrial dynamics, mitophagy, and other mitochondrial proteostasis mechanisms such as mitochondrial unfolded protein response (mtUPR) or mitochondrial-derived vesicles (MDVs), to ensure that the necessary number of functional mitochondria maintains the demands and function of the cell [3, 5]. Mitochondrial biogenesis plays an important role in mitochondrial quality control by creating new mitochondria to replace damaged mitochondria [6]. Mitochondrial dynamics include both mitochondrial division and mitochondrial fusion, which are critical for maintaining mitochondrial homeostasis and normal function [7, 8]. Selective degradation of mitochondria, known as mitophagy, is an important mitochondrial quality control mechanism that aims to control and remove defective mitochondria from the cell. In mammals, different mitophagy effectors, including the mitophagy receptors NIX, BNIP3, and FUDNC1 and the PINK1/Parkin pathway, have been identified to participate in the selective clearance of mitochondria [9]. One common feature of mitophagy receptors is that they harbor an LC3-interacting region (LIR) that interacts with LC3, thus promoting the sequestration of mitochondria into the isolation membrane [10]. Mitophagy often takes place under baseline conditions. For example, NIX can remove mitochondria from the mature erythrocytes during development, and Parkin and the mitochondrial E3 ubiquitin protein ligase 1 (MUL1) are necessary for the degradation of paternal mitochondria after fertilization in mice [11]. In response to severe stress, several molecules, including PINK1/Parkin, NIX/BNIP3 and FUNDC1, were found to participate in selective mitophagy [10, 12]. The subcellular localization of the mitophagy-related proteins are summarized in the following Table 1. PINK1/Parkin-mediated mitophagy is involved in the selective removal of damaged mitochondria that have lost their membrane potential [13]. In response to hypoxia, both NIX/BNIP3 and FUNDC1 are involved in the subsequent induction of mitophagy [14]. Indeed, mitophagy has been recently proposed to play critical roles in different diseases [15]. However, it is currently unknown whether there is a relationship between mitophagy and various types of cell death and whether mitophagy can be used to serve as a potential therapeutic target by regulating the cell death pathway. Herein, we discuss mitophagy in different physiological and pathological contexts and then relate it to cell death pathways (necrosis, apoptosis, pyroptosis, ferroptosis, and necroptosis) (Figure 1). Understanding how mitophagy regulating the cell death pathway contributes to the maintenance of mitochondrial homeostasis could provide insights into the development of targeted treatments when these systems fail in disease.

## 2. Mitophagy

Mitophagy is a special form of autophagy, which regulates mitochondrial turnover. The term “mitophagy” was first in use by Scott in 1998 and was first proposed as the nonrandom nature of the process by Lemasters in 2005 [16, 17]. There are at least two major mechanisms by which mitochondria are targeted for mitophagy: ubiquitin-mediated and transmembrane receptor-mediated [18–20]. PTEN-induced putative kinase 1- (PINK1-) mediated mitophagy is a selective process in that mitochondria first depolarize after mitochondrial damage induced by various factors such as reactive oxygen species (ROS) or mitochondrial DNA damage, and then damaged mitochondria may recruit autophagy receptor for autophagosome formation around mitochondria and fused with lysosomes, thus suffering from the degradation process [21]. Because of the danger of damaged or aged mitochondria in the cell, the timely elimination of abnormal mitochondria through mitophagy is essential for maintaining mitochondrial balance and the integrity of the cell [10, 12]. For example, neurons are strictly dependent on mitochondrial ATP production, and the accurate and proper degradation of dysfunctional mitochondria by mitophagy is essential for maintaining control over mitochondrial quality and quantity in neurons [22–24]. Wang et al. reported that mitophagy induced by sigma-1 receptor agonist in dopaminergic neurons exerts a neuroprotective effect in the MPTP-induced mouse model of Parkinson's disease [25]. There has been rapid progress in the study of mitophagy in the past few years, which has led to a greater understanding of the molecular regulation mechanisms of mitophagy in diseases.

### 2.1. The Signaling Pathways of Mitophagy

#### 2.1.1. PINK1-Parkin Signaling Pathway in Mitophagy

The PINK1-parkin pathway is considered to be the most classic pathway that causes mitophagy. PINK1 is a mitochondrial Ser/Thr kinase that was recognized in Parkinson's disease in 2004 [26]. Under basal conditions, PINK1's N-terminus is transferred across the outer mitochondrial membrane (OMM) to the inner mitochondrial membrane (IMM), with the kinase domain located closer to the C-terminus protruding out into the cytosol [27]. PINK1 undergoes a series of degradation processes by matrix processing peptidases (MPP) in the mitochondrial matrix, presenilin-associated rhomboid-like (PARL) in the mitochondrial inner membrane, and proteases in the cytoplasm, which leads to low levels of PINK1 in healthy cells [28]. However, in response to mitochondrial stressors, such as membrane depolarization, mitochondrial complex dysfunction, mutagenic stress, and proteotoxicity, PINK1 accumulates on the OMM of injured mitochondria by impairing intermembrane transport of the N-terminus domain to the IMM [29, 30]. Due to PINK1's ability to rapidly accumulate on the OMM and activate in response to mitochondrial stressors, it has been suggested that PINK1 functions as a sensor to probe for damaged mitochondria [31, 32]. Recently, it was also reported that PINK1-s (a short form of PINK1), as a sensor of proteasomal activities, protected cells against proteasome stress through inhibiting protein synthesis [33, 34]. Subsequent homodimerization of PINK1 on the OMM leads to autophosphorylation, which promotes kinase activation and facilitates the translocation of Parkin to the membrane of damaged mitochondria and recruitment of substrates ubiquitin to the mitochondria [13, 27, 35]. PINK1 activates Parkin through two mechanisms. First, it phosphorylates ubiquitin on S65, which competes with an autoinhibitory domain within Parkin and stabilizes it in an active conformation. Second, PINK1 directly phosphorylates Parkin on S65 in Parkin's ubiquitin-like domain, which induces conformational changes that allow for the activation of Parkin activity [36–40]. Subsequently, mitochondria-localized Parkin ubiquitinates a wide variety of cytosolic and outer mitochondrial membrane proteins, which may either be degraded through the proteasome or serve as binding partners for autophagic receptors that contain the LIR domain for the direct interaction with LC3 to recruit autophagosomal membranes to the mitochondria for the formation of the autophagosome [41–44]. PINK1 and Parkin can also function independently in the mitophagy pathway. For example, it has been shown that PINK1 can recruit the autophagy receptor nuclear dot protein 52 kDa (NDP52) and optineurin (OPTN) directly to mitochondria to activate mitophagy independent of Parkin [45]. The authors also showed that NDP52 and OPTN recruit Unc-51 like autophagy activating kinase 1 (ULK1) to mitochondria to induce mitophagy independent of Parkin. Igarashi et al. reported that gemcitabine treatment induces a novel type of PINK1-dependent Parkin-independent mitophagy, where mitochondrial ubiquitin ligase (MUL1) facilitates PINK1 stabilization [46]. Since Parkin is not expressed in HeLa cells, these chemicals induce mitophagy through a parkin-independent pathway. However, the specific mechanism for the activation of downstream factors of PINK1 and gemcitabine-induced mitophagy remains unclear. Kubli et al. discovered that mitochondrial translocation of Parkin and subsequent activation of mitophagy occur independently of PINK1 in myocytes [47]. This is unexpected since most studies have reported that accumulation of PINK1 on the outer mitochondrial membrane is required for recruitment of Parkin [48, 49]. It is possible that other kinases can compensate for the lack of PINK1 in the cell. However, the specific mechanism for the activation of parkin independently of PINK1 remains unclear.

#### 2.1.2. BNIP3 and NIX/BNLP3L Signaling Pathway in Mitophagy

BNIP3 (BCL2 and adenovirus E1B 19-kDa-interacting protein 3) and NIX (Nip3-like protein X, also known as BNIP3L) are bifunctional mitochondrial proteins: they contain a BH3 domain with homology to the BH3-only proapoptotic members of the Bcl-2 family proteins and thus can induce cell death [50–52]. Under physiological conditions, the BNIP3 protein is generally expressed in normal cells and tissues at a low level. Various stresses, particularly hypoxia, can induce BNIP3-mediated mitophagy since BNIP3 is transcriptionally activated by the transcription factor hypoxia-inducible factor 1(HIF-1) under hypoxia, which is why it is extremely sensitive to hypoxia and is generally used as a typical target gene of HIF-1 [14, 53]. BNIP3 is found to interact with LC3 family proteins via their LIR motifs facing the cytosol, and the enhanced interaction between mitochondria-localized BNIP3 with autophagosome-localized LC3 may subsequently contribute to the induction of excessive mitophagy [54–56]. A recent study identified Ser17 and Ser24 as phosphorylation sites of BNIP3 that are responsible for enhancing the BNIP3–LC3 interaction [57]. Despite the bulk of relevant research concerning BNIP3's involvement in dysfunctional mitochondria, the regulatory mechanisms of BNIP3 in mitophagy are still poorly understood. Madhu et al. reported that the hypoxic regulation of mitophagy in nucleus pulposus cells is dependent on the HIF-1*α*-BNIP3 axis [58]. Mitochondrial disease is difficult to diagnose because it affects different people in different ways. Secondary mitochondrial dysfunction (SMD), which is different from primary mitochondrial disease (PMD), can be caused by genes encoding neither function nor production of specific proteins, oxidative stress, drug toxicity, or environmental factors [59, 60]. Distinguishing whether mitochondrial dysfunction is inherited or acquired is extremely challenging. The regulatory mechanisms of BNIP3 actions in mitophagy could lead to the development of novel therapeutic platforms for SMD [50]. Jin et al. reported that the HIF-1*α*/BNIP3 signaling pathway is associated with BDNF/TrkB-induced mitophagy in brain microvascular endothelial cell injury [61]. A valuable article pointed out that BNIP3-mediated mitophagy protects SH-SY5Y neuroblastoma cells against TNF*α*-induced inflammatory injury [62]. O'Sullivan et al. revealed the functional importance of BNIP3-mediated mitophagy in promoting the generation of natural killer cell memory [63].

The subcellular locations of NIX are on the outer mitochondrial membrane and endoplasmic reticulum. It is reported that NIX is involved in programmed removal of mitochondria in immature red blood cells, and reticulocytes Nix functions as a regulated mitophagy receptor [64, 65]. In addition, NIX-mediated mitophagy also plays an indispensable role in regulating platelet mitochondria quality/activity. Zhang et al. reported that Nix-mediated mitophagy regulates platelet activation and life span [66]. Like BNIP3, NIX also has key domains that can directly bind to Atg8/LC3 in the family-LIR domain as a receptor for autophagy machinery [64]. In this regard, phosphorylation of serine residues flanking the NIX LIR regulates its interaction with LC3 and mitophagy [67]. Moreover, the previous study has demonstrated that Nix is readily phosphorylated by Casein Kinase 2 at Ser117, Ser118, and Ser120 in HEK 293 T cells, suggesting that a specific kinase may play a role in the activation of Nix-mediated mitophagy through phosphorylation of Nix [68–70]. Melser et al. proposed that GTPase Ras homolog enriched in brain protein (Rheb) is recruited to the OMM to facilitate the interaction between Nix and LC3, thus promoting the engulfment of mitochondria by nascent autophagosomes [71]. Generally, three possible working models of Nix in mitophagy induction are as follows. Firstly, Nix can serve as a receptor for autophagy machinery by interacting with Atg8 family through its LIR motif. Secondly, Nix can interplay with Parkin or Rheb, important proteins for mitophagy, to initiate mitophagy. Finally, as a BH3-only protein, Nix can compete with Beclin-1 to bind other members of the Bcl-2 family resulting in increased free Beclin-1 in the cytosol, which further promotes autophagy flux [72].

#### 2.1.3. FUNDC1 Signaling Pathway in Mitophagy

Fun14 domain-containing protein 1 (FUNDC1), a protein localized in the outer mitochondrial membrane, is considered as another novel receptor that involves the process of mitophagy [73]. FUNDC1-mediated mitophagy, involved in hypoxia-induced mitophagy, is different from the Pink-Parkin mitophagy pathway since knockdown of Parkin does not prevent mitochondrial degradation and LC3-I to II transition in response to hypoxia [74]. Consistent with NIX and BNIP3, the main characteristic is their conserved LC3 interaction region (LIR) with a W/Y/FxxL/I/V motif, which can interact with LC3, a biomarker protein of the autophagosome in mammalian cells [75]. The interaction between LC3B and FUNDC1 is dramatically affected by the phosphorylation states of FUNDC1. Several reports have revealed that FUNDC1 regulates hypoxia-induced selective mitophagy through reversible phosphorylation at several critical sites including Tyr18, Ser13, and Ser17 [73, 76]. Under normal circumstances, FUNDC1 interacts with LC3B through its classical LIR-Y(18)xxL(21), while hypoxia-induced dephosphorylation of FUNDC1 enhanced its interaction with LC3 for selective mitophagy. It was identified that the phosphoglycerate mutase family member 5 (PGAM5), a serine/threonine phosphatase located at mitochondria, can interact with and then dephosphorylate LIR motif of FUNDC1 at Ser13 upon FCCP treatment or hypoxia stimulation [77, 78]. FUNDC1-related mitochondrial dysfunction contributes to various pathophysiological processes, such as heart diseases, intestinal ischemia/reperfusion injury, and metabolic disorders. FUNDC1 is generally considered to be protective in these diseases because FUNDC1-mediated mitophagy can alleviate the damage caused by intracellular stress such as hypoxia and thus benefit overall outcomes [79–81]. FUNDC1 is also localized in mitochondrial-associated endoplasmic reticulum membranes (MAMs) and plays a significant role in the communication between the ER and mitochondria in the heart and sustains normal cardiac function [82].

#### 2.1.4. Other Signaling Pathway in Mitophagy

Recent evidence indicates that activating molecule in BECLIN1-regulated autophagy (AMBRA1) plays a role in mitophagy [83]. Strappazzon et al. reported that a pool of AMBRA1 is already docked at mitochondria by BCL-2 under normal conditions. This reserve of AMBRA1 is released from BCL-2 when autophagy is induced, allowing it to bind to BECLIN1 and initiate the formation of the phagophore at the mitochondrion [84, 85]. Cen et al. report that MCL-1, an important anti-apoptotic protein, is a mitophagy receptor that can be targeted to induce mitophagy and identify MCL-1 as a drug target for therapeutic intervention in Alzheimer's disease [86].

### 2.2. Interaction between Pathways of Mitophagy

We briefly introduced the pathways that cause mitophagy. A lot of indepth research also confirmed that distinct mitophagy pathways cooperate to regulate mitochondrial homeostasis [56]. For example, in the presence of PINK1/Parkin-mediated mitophagy, Nix seems to function downstream of Parkin as a substrate, then is ubiquitinated by Parkin, and binds to LC3/GABARAP through interaction with the neighbor of BRCA1 [69, 95]. In addition, BNIP3 is able to inhibit PINK1 proteolytic degradation and stabilize PINK1 on OMM to facilitate Parkin mitochondrial recruitment and mitophagy [96, 97]. Upon mitochondrial depolarization, the Bcl-2 family member Mcl-1 underwent rapid Parkin- and PINK1-dependent polyubiquitination and degradation, which sensitized toward apoptosis via opening the Bax/Bak channel [98]. AMBRA1-mediated mitophagy is dependent on Parkin, and the overexpression of AMBRA1 in Parkin-/- cells fails to restore mitophagy levels [99]. These findings suggest that these pathways cooperate with each other to ensure efficient mitophagy.

## 3. Mitophagy and Cell Death

### 3.1. Mitophagy and Necrosis

Cell death can be classified into two major types: programmed cell death (PCD) and necrosis in accordance with its morphological appearance [100]. Necrosis has been characterized as passive, accidental cell death resulting from environmental perturbations with the uncontrolled release of inflammatory cellular contents [101]. The change of the cell nucleus is the main morphological sign of necrosis. Under the light microscope, there are three types: nuclear shrinkage, nuclear fragmentation, and nuclear dissolution. The nucleus staining of fluorescent dyes such as DAPI is significantly reduced when staining the nucleus of necrotic cells. The cell membrane loses the integrity of the membrane, releasing extracellular content, leading to activation of the immune system and extensive inflammation [102]. The occurrence of mitophagy is closely related to necrosis, which has different effects on different stages of the disease [103, 104]. The immoderacy of mitophagy may eliminate too many mitochondria so that cells cannot maintain basic functions and undergo cell necrosis [105, 106]. Paech et al. reported that mitochondrial repair by mitophagy, which can be regarded as a protective process to remove damaged mitochondria, is impossible, and cells exposed to the kinase inhibitors will undergo necrosis when the toxic insult is too pronounced [107]. Intracellular ATP levels have a determining role in the interplay between apoptosis and necrosis: high ATP levels typically enable a cell to undergo apoptosis, whereas low ATP levels favor necrosis [108–110]. Xia et al. reported that mitophagy switches cell death from apoptosis to more efficient necrosis due to persistent ATP consumption and exhaustion along with viral replication in non-small cell lung cancer cells (NSCLCs) following measles virus vaccine strain Edmonston B infection [111]. Interestingly, mitophagy was accompanied by increased necrosis. Vande Velde et al. propose that BNIP3 is a gene that mediates necrosis-like cell death through mitochondrial permeability transition pore (mPTP) opening and mitochondrial dysfunction [112]. Dhingra et al. report that BNIP3 mediates doxorubicin-induced cardiac myocyte necrosis and mortality through changes in mitochondrial respiratory chain signaling [113].

### 3.2. Mitophagy and Apoptosis

In 1972 Kerr, Wyllie and Currie published a paper describing a type of cell death that is distinct from necrosis and named apoptosis [114]. This is the first time apoptosis has been presented to the world [115–117]. Apoptosis is considered to be an important part of various processes under physiological conditions, including normal cell replacement, normal development, and function of the immune system. In addition, the production of apoptotic cells can be found under pathological conditions [118]. Apoptosis is a form of programmed cell death (PCD) that occurs in multicellular organisms. It causes a series of continuous reactions by causing the activation of various types of cysteine-aspartic proteases in the cell, which ultimately leads to cell death. In this process, the characteristics of apoptosis lie in the formation of apoptotic bodies. Apoptotic bodies containing the contents of dead cells can be engulfed by surrounding cells; so, the occurrence of apoptosis will not cause leakage of the contents and cause damage to surrounding cells [119]. Apoptosis is composed of two internal and external pathways [120]. The internal pathway is also called the mitochondrial pathway of apoptosis, and the external pathway is the death receptor pathway of apoptosis [115]. A large number of studies have been conducted on these two pathways, and many key apoptotic proteins have been identified, such as caspase3.

Through mitophagy, cells are able to cope with mitochondrial stress until the damage becomes too great, which leads to the activation of apoptosis [121, 122]. When there is vast mitochondrial damage, such as that which occurs during myocardial infarction, activation of apoptotic proteases will shut down mitophagy and activate apoptosis to ensure cell death [84]. Moreover, mitophagy exerts a significant neuroprotective effect against Mn-induced apoptosis in dopaminergic SH-SY5Y cells [123]. Ham et al. found that the PINK1–Parkin pathway controls both mitophagy and apoptosis by inducing two different types of ubiquitination on VDAC1. The polyubiquitinated VDAC1 promotes Parkin-mediated mitophagy, whereas the monoubiquitinated VDAC1 inhibits mitochondrial calcium uptake, which ultimately protects cells from apoptosis [42]. When Parkin induces polyubiquitination on VDAC1, the ubiquitinated VDAC1 triggers Parkin-mediated mitophagy by recruiting p62/sequestosome1 (SQSTM1) and LC3B to the mitochondria [41]. Oligomeric assembly of VDAC1 was shown to be coupled to apoptosis induction. It may be possible that the monoubiquitination disturbs VDAC1 self-oligomerization, resulting in the prevention of OMM permeabilization and inhibition of apoptosis [42, 124, 125]. Previous studies have reported that mitophagy is a defense mechanism to support cancer survival in response to acute or chronic stress. Wei et al. found that the decreased expression of PINK1/Parkin induced by matrine (a natural alkaloid extracted from the roots of Sophora favescens) leads to the inhibition of mitophagy, which ultimately leads to increased apoptosis. When PINK1 is overexpressed, mitophagy can be reactivated and apoptosis reduced with the activation of caspase-9 [126]. In contrast, excessive abnormal mitophagy exacerbates apoptosis [127]. Chen et al. reported that ketoconazole exacerbates mitophagy to induce apoptosis by downregulating cyclooxygenase-2 in hepatocellular carcinoma [128]. Most of the above described molecular mechanisms involved in the mitophagic processes have been shown to be dysregulated in cancer, but whether they behave as tumor promoter or tumor suppressor seems to be highly dependent on the cancer subtype and context. Moreover, more mechanistic studies are necessary to understand the extent of the relationship between cancer-associated mitophagy and healthy tissue as well as the mitophagic pathways involved. A tight control of the mitochondrial network homeostasis is essential for cancer cells. Therefore, abnormal mitophagy is thought to promote apoptosis by disrupting mitochondrial homeostasis.

### 3.3. Mitophagy and Pyroptosis

Pyroptosis is a type of programmed cell death that features pore formation on the plasma membrane, cell swelling, and plasma membrane disruption, similar to that of necrosis but not apoptosis [101, 129]. It has been reported that the most important executor of pyroptosis is gasdermin D (GSDMD); so, pyroptosis is also considered to be gasdermin-mediated programmed cell death [130]. Pyroptosis is thought to be initially triggered by nucleotide-binding oligomerization domain-like receptors (NLRs) under disease conditions, which is mainly related to the formation of inflammatory bodies, especially NLR family pyrin domain containing 3 (NLRP3) and apoptosis-associated speck-like protein containing a CARD protein (ASC). The formation of inflammatory bodies recruits and activates caspase 1, prompting a series of reactions, which ultimately leads to the cleavage of GSDMD. The inflammasome-activated gasdermin-N domain of GSDMD can be made in liposomes containing phosphatidylinositol or cardiolipin or a mixture of natural polar lipids. A wide range of pores are formed on the liposomes, and the formation of GSDMD pores in the membrane disrupts the osmotic potential, causing the cells to swell and eventually dissolve. The overflow of cell contents also causes an increased inflammatory response to the surroundings. In addition to caspase 1, other inflammatory proteases have also been shown to be closely related to the occurrence of pyroptosis, including caspase 11, 4, and 5. They can induce the downstream GSDMD cleavage and then cause pyroptosis; so, it is also listed as one of the pyroptosis ways [130, 131].

Although the mechanisms underlying the relationship between mitophagy and pyroptosis are complex, increasing studies have reported that mitophagy plays an important function in the regulation of pyroptosis. Yu et al. reported that caspase-1–mediated cleavage of parkin, a key mitophagy regulator, contributes to inflammasome-mediated block of mitophagy and increased mitochondrial damage augments pyroptosis [132]. Yu et al. found that liraglutide (glucagon-like peptide-1 receptor agonist) inhibits the activation of NLRP3 inflammasome and pyroptosis by activating Pink1/Parkin-mediated mitophagy to improve nonalcoholic steatohepatitis. After simultaneous use of autophagy inhibitor 3-Methyladenine (3-MA) or siRNA against PINK1, mitophagy was inhibited, but NLRP3 inflammatory corpuscle activation and GSDMD expression were reversed [133]. Besides, other studies have indicated that palmatine inhibits LPS/ATP-induced NLRP3 activation, which can be reversed by mitophagy inhibitors or PINK1-siRNA [134]. Blocking Parkin-mediated mitophagy restored the sensitivity of HIV-productively infected astrocytes to pyroptosis, suggesting that mitophagy is required for pyroptosis resistance, thus favoring cell survival in HIV-infected astrocytes [135]. In addition to pink1/parkin, NIX-mediated mitophagy plays a certain role in the occurrence and the regulation of pyroptosis. Peng et al. reported that the silenced NIX expression in murine macrophage cell, active caspase-1, and mature interleukin-1*β* expression levels was increased and LC3 was reduced, suggesting that NIX inhibits macrophage pyroptosis via mitophagy [136].

### 3.4. Mitophagy and Ferroptosis

Ferroptosis is a new type of cell death that was discovered in recent years and is usually accompanied by a large amount of iron accumulation and lipid peroxidation during the cell death process [137, 138]. Ferroptosis, an iron-dependent form of regulated necrosis, has emerged as a new cell death highly relevant to disease [139]. It is typically characterized in the morphological analysis by the presence of smaller than normal mitochondria, along with condensed mitochondrial membrane densities, reduction or vanishing of mitochondria crista, and outer mitochondrial membrane rupture [140]. Ferroptosis refers to the oxidative cell death that occurs under the induction of small molecules and is iron ion-dependent. Its occurrence is caused by the imbalance between the production and degradation of reactive oxygen species in the cell [141]. Ferroptosis inducers act directly or indirectly on glutathione peroxidase (GPXs) through different pathways, resulting in decreased antioxidant capacity of cells, accumulation of ROS, and ultimately oxidative death of cells [142].

The role of mitophagy in ferroptosis has received very little attention thus far, limited mainly to in vitro studies using cancer cells. In HT1080 cells (fibrosarcoma) treated with carbonyl cyanide 3-chlorophenyl hydrazine (CCCP), Parkin-mediated mitophagy inhibits cysteine-deprivation-induced ferroptosis [139]. The escalated ROS generation in cancer cells contributes to the biochemical and molecular changes necessary for the tumor initiation, promotion, and progression. However, elevating ROS to highly toxic levels intracellularly, thereby activating various ROS-induced cell death pathways, may provide a unique opportunity to eliminate cancer cells ([143]). In melanoma cells, the inhibitors of mitochondrial complex I, BAY 87-2243, can promote the increase of mitophagy-dependent ROS, leading to ferroptosis. Knockdown of PINK1 inhibited the BAY 87-2243-induced *Δψ* depolarization, mitophagy stimulation, ROS increase, and ferroptosis in cells [144, 145]. In breast cancer and glioblastoma cell lines, BAY 11-7085 (inhibitor of NK-*κ*B activation) induced ferroptosis via nuclear factor-E2-related factor 2- (Nrf2-) solute carrier family 7 membrane 11 (SLC7A11)—heme oxygenase-1 (HO-1) pathway and causes compartmentalization of HO-1 into the nucleus and mitochondria, and followed mitochondrial dysfunctions, leading to lysosome targeting for mitophagy [146]. The abovementioned studies in cancer cells provide empirical in vitro support for the antiferroptotic action of mitophagy. However, direct in vivo evidence for an antiferroptotic effect of mitophagy induction does not yet exist, and it is currently unknown whether and to what extent the demonstrated efficacy of mitophagy activation in other disease models involve attenuation of ferroptosis.

### 3.5. Mitophagy and Necroptosis

Necroptosis is the best-characterized form of programmed necrosis, showing features of both apoptosis and necrosis [147]. Base on the driving factors, necroptosis is classified into three categories: (1) extrinsic necroptosis is stimulated by tumor necrosis factor-alpha (TNF*α*), (2) intrinsic necroptosis is stimulated by reactive oxygen species (ROS), and (3) ischemia mediated intrinsic necroptosis [148]. Receptor-interacting serine/threonine-protein kinase 1 (RIPK1) is a member of the TNF receptor 1, a ubiquitous membrane receptor that binds TNF*α* [149]. RIPK1 is a 76 kDa protein with an amino-terminal (N-terminal) kinase domain, a carboxy-terminal (C-terminal) death domain, and an intermediate domain with a receptor-interacting protein homotypic interacting motif (RHIM) that can bind to other RHIM-containing proteins [150, 151]. The serine/threonine kinase RIPK1 and receptor-interacting serine/threonine-protein kinase 3 (RIPK3) are the core components of the necroptotic signaling platform [152]. In classical necroptosis, necrosome complex is formed due to interaction between RIPK1 and RIPK3 through the RHIM domain. The necrosome inhibits the adenine nucleotide translocase in mitochondria to decrease cellular ATP levels [153]. A role for mitochondria in necroptosis has been proposed, in large part, by its association with a ROS burst [144, 154, 155]. Tait et al. reported that elimination of mitochondria prevents necroptosis-associated ROS production, but does not alter RIPK3-dependent cell death [156]. It is not known whether mitophagy can regulate necroptosis. The mixed-lineage kinase domain-like (MLKL) protein is phosphorylated by RIP3 at Thr357 and Ser358 residues, and these events are critical for the execution of necroptosis [152]. Mizumura et al. reported that cigarette smoke exposure-induced MLKL phosphorylation was decreased in PINK1-knockdown cells, suggesting that PINK1-dependent mitophagy can act as an upstream regulator of the necrosome [157, 158]. Overall, the importance of mitochondria during necroptosis appears to be cell type- and stimulus-dependent.

## 4. Conclusion

Understanding the interface between mitophagy and cell death is important for understanding the pathogenesis of the disease. Basal levels of mitophagy are important for maintaining cellular homeostasis and protecting cells against the accumulation of dysfunctional mitochondria. There is also a crosstalk between mitophagy and cell death pathways. The manipulation of proteins that regulate mitochondrial integrity and mitophagy represents future therapeutic targets to preserve cell viability and prevent the development of the disease. Therefore, it is important to gain insights into the mechanisms regulating the balance between mitophagy and death, both under normal conditions and in diseased states. To date, only a few studies have been performed on this project. In the future, our lab will pay more attention to seek the inner relationship between mitophagy and cell death pathways.

## Figures and Tables

**Table 1:** The subcellular localization of the mitophagy-related protein.

Protein	Subcellular localization	References
PINK1	(1) Mitochondria (main): the inner and outer mitochondrial membrane (2) Cytosol (3) Endoplasmic reticulum	([87]; [88]; [89]; [90])
Parkin	(1) Cytosol (main) (2) Dysfunctional mitochondria (3) Endoplasmic reticulum (4) Golgi apparatus	[88] [29] [91] [92]
NIX/BNIP3L	(1) Mitochondria: the outer mitochondrial membrane (2) Endoplasmic reticulum	[51, 93] [66]
BNIP3	(1) Mitochondria: the outer mitochondrial membrane (2) Endoplasmic reticulum	[51] [94]
FUNDC1	(1) Mitochondria: the outer mitochondrial membrane	[75]

Note: PINK1: PTEN-induced putative kinase 1; FUNDC1: Fun14 domain-containing protein 1; BNIP3L: BCL2/adenovirus E1B 19 kDa protein-interacting protein 3-like; BNIP3: BCL2-interacting protein 3.
